# Alterations in glymphatic system and brain morphology in patients with temporal lobe epilepsy

**DOI:** 10.1186/s12880-026-02279-2

**Published:** 2026-03-13

**Authors:** Xinming Huang, Meifeng Wang, Zhenxing Wu, Rifeng Jiang, Wanhui Lin, Chunnuan Chen, Yunjing Xue, Yì Xiáng J. Wáng

**Affiliations:** 1 Department of Radiology, Fujian Medical University Union Hospital, No. 29 Xinquan Road, Fuzhou, Fujian 350001 China; 2 Department of Neurology, The Second Affiliated Hospital, Fujian Medical University, No. 950 Donghai Street, Quanzhou, Fujian Province 362000 China; 3 Department of Neurology, Fujian Medical University Union Hospital, Fujian, China; 4 Rare Disease Medical Center, The Second Affiliated Hospital, Fujian Medical University, Quanzhou, Fujian Province 362000 China; 5 Department of Imaging and Interventional Radiology, Faculty of Medicine, The Chinese University of Hong Kong, Shatin, New Territories, Hong Kong SAR China

**Keywords:** Temporal lobe epilepsy, Diffusion tensor imaging analysis along the perivascular space, Diffusion kurtosis imaging analysis along the perivascular space, Glymphatic system, Morphology

## Abstract

**Background:**

Although the diffusion tensor imaging analysis along the perivascular space (DTI-ALPS) method has been widely applied to gauge glymphatic function in temporal lobe epilepsy (TLE), glymphatic microstructure remains underexplored. Furthermore, the choroid plexus and ventricles are key elements of the brain clearance network. This study aimed to comprehensively evaluate changes in ALPS indices alongside hippocampal, choroid plexus, and lateral ventricular volumes in unilateral TLE.

**Methods:**

We recruited 14 healthy controls (HCs) and 30 TLE patients, including 16 left TLE (LTLE) and 14 right TLE (RTLE) patients. We evaluated differences in ALPS indices and morphological metrics, including hippocampal volume/total intracranial volume (HV/TIV), choroid plexus volume/TIV (CPV/TIV), lateral ventricular volume/TIV (LVV/TIV), and their asymmetry indices (AIs), between TLE patients and HCs. Furthermore, we assessed correlations among ALPS indices, morphological metrics, and clinical characteristics in TLE patients.

**Results:**

Compared to HCs’ bilateral means, TLE patients exhibited significantly decreased ipsilateral HV/TIV (*p* = 0.003); both ipsilateral and contralateral CPV/TIV (*p* = 0.039 and *p* = 0.042 respectively) and LVV/TIV (*p* = 0.030 and *p* = 0.032 respectively) were significantly increased; both the ipsilateral DTI-ALPS and diffusion kurtosis imaging analysis along the perivascular space (DKI-ALPS) indices were significantly decreased (*p* = 0.011 and 0.008 respectively). In LTLE patients, only AI of the DKI-ALPS index showed a significant increase (*p* < 0.001). Within the RTLE group, AI of HV/TIV correlated with both onset age and disease duration (*p* = 0.009 and *p* = 0.008 respectively). In the TLE cohort, correlations were observed between onset age and both ipsilateral HV/TIV and AI of CPV/TIV (both *p* < 0.05). Furthermore, AI of HV/TIV was positively associated with disease duration (*p* = 0.011), and AI of LVV/TIV was positively linked to seizure frequency (*p* = 0.032).

**Conclusions:**

Our study demonstrated alterations beyond hippocampal atrophy in TLE, involving the brain waste clearance network. LTLE and RTLE might exhibit distinct alteration patterns. These findings could provide new insights into TLE pathogenesis.

**Supplementary Information:**

The online version contains supplementary material available at 10.1186/s12880-026-02279-2.

## Background

Temporal lobe epilepsy (TLE) is the most common form of focal epilepsy in adults, with hippocampal sclerosis (HS) representing its most prominent histopathological hallmark [1]. Beyond hippocampal pathology, glymphatic system dysfunction has emerged as a contributing mechanism in epilepsy [2]. The glymphatic system primarily comprises periarterial and perivenous spaces, along with aquaporin-4 (AQP4) water channels expressed on astrocytic endfeet surrounding blood vessels [3]. Under physiological conditions, it facilitates metabolite clearance through perivascular inflow of cerebrospinal fluid (CSF), exchange with interstitial fluid (ISF), and perivenous outflow [4], thereby removing soluble waste and metabolic byproducts [5]. Given that ISF stagnation induces retrograde expansion of perivascular spaces (PVS), and enlarged PVS (EPVS) is increasingly recognised as a potential indicator of glymphatic dysfunction [6, 7]. Research demonstrated that patients with high temporal lobe EPVS burden tend to exhibit higher pretreatment seizure density and reduced likelihood of delayed seizure freedom attainment [6]. Thus, glymphatic impairment may represent a novel pathomechanism in TLE. Further investigation into glymphatic functional changes in TLE patients could provide critical insights into this disorder.

Prior glymphatic system research relies on invasive tracers or animal models [8–10], whereas the diffusion tensor imaging analysis along the perivascular space (DTI-ALPS) method enables non-invasive assessment in vivo [11]. This method evaluates water molecule movement along perivascular spaces by measuring the diffusivity around medullary veins at the lateral ventricular level, indirectly reflecting individual glymphatic activity [11]. Furthermore, the DTI-ALPS index has demonstrated significant correlation with the brain glymphatic clearance rate measured by classical glymphatic magnetic resonance imaging (MRI) with intrathecal gadolinium-based contrast administration, exhibiting excellent inter-scanner reproducibility, inter-rater reliability and test-retest repeatability [12–14]. Numerous studies on TLE have successfully implemented the DTI-ALPS method. Research indicated significantly reduced ipsilateral DTI-ALPS indices of TLE patients compared to both the contralateral indices of the patients and the bilateral means of the healthy controls (HCs), with notable increases following successful anterior temporal lobectomy [15, 16]. Concurrently, significant correlations have been reported between the ipsilateral DTI-ALPS index and disease duration in TLE patients [15]. Furthermore, left TLE (LTLE) and right TLE (RTLE) patients might exhibit distinct functional alteration patterns of the glymphatic system [17]. However, the inherent limitations of diffusion tensor models in accurately describing diffusion behaviour [18] highlight the urgent need for more comprehensive biomarkers to validate glymphatic system alterations in TLE patients. Notably, diffusion kurtosis models have the superiority in resolving diffusion information of the crossing fibre regions. Therefore, the ALPS index was recently proposed to calculate using diffusion kurtosis imaging (DKI), namely the diffusion kurtosis imaging analysis along the perivascular space (DKI-ALPS) index. Unlike the DTI-ALPS index, which is a marker of glymphatic function, the DKI-ALPS index offers complementary information on the glymphatic system’s microstructural complexity, which demonstrated more significant alterations than functional measures in migraine patients [19]. However, the efficacy of DKI-ALPS in TLE patients remains unclear.

Beyond the glymphatic system, critical morphological components of the brain waste clearance network include the choroid plexus (CP) and ventricle. Research indicated that decreased DTI-ALPS index exhibited significant correlation with increased choroid plexus volume (CPV), both serving as independent risk factors for semantic fluency impairment in TLE patients [20]. Furthermore, scholars have documented increase in ipsilateral lateral ventricular volume (LVV) in patients with mesial temporal lobe epilepsy and hippocampal sclerosis [21]. However, the correlation between the glymphatic system function and morphology including CPV and LVV remains unclear. Functional alterations of the brain waste clearance network in TLE await further elucidation.

Based on the aforementioned evidence, we hypothesized that patients with unilateral TLE would exhibit glymphatic dysfunction, altered glymphatic microstructure, and morphological changes in key brain structures (hippocampi, choroid plexuses, and lateral ventricles). We further postulated potential associations between these glymphatic and structural alterations. Considering that the DKI-ALPS index may provide complementary information on microstructural integrity compared to the function-oriented DTI-ALPS index, this study aimed to: (1) explore alterations in ALPS indices and structural volumes, (2) investigate the roles of ALPS indices in understanding glymphatic system changes, (3) assess correlations between ALPS indices and morphological characteristics, and (4) examine associations between these imaging metrics and clinical characteristics.

## Methods

### Participants

**Fig. 1:**
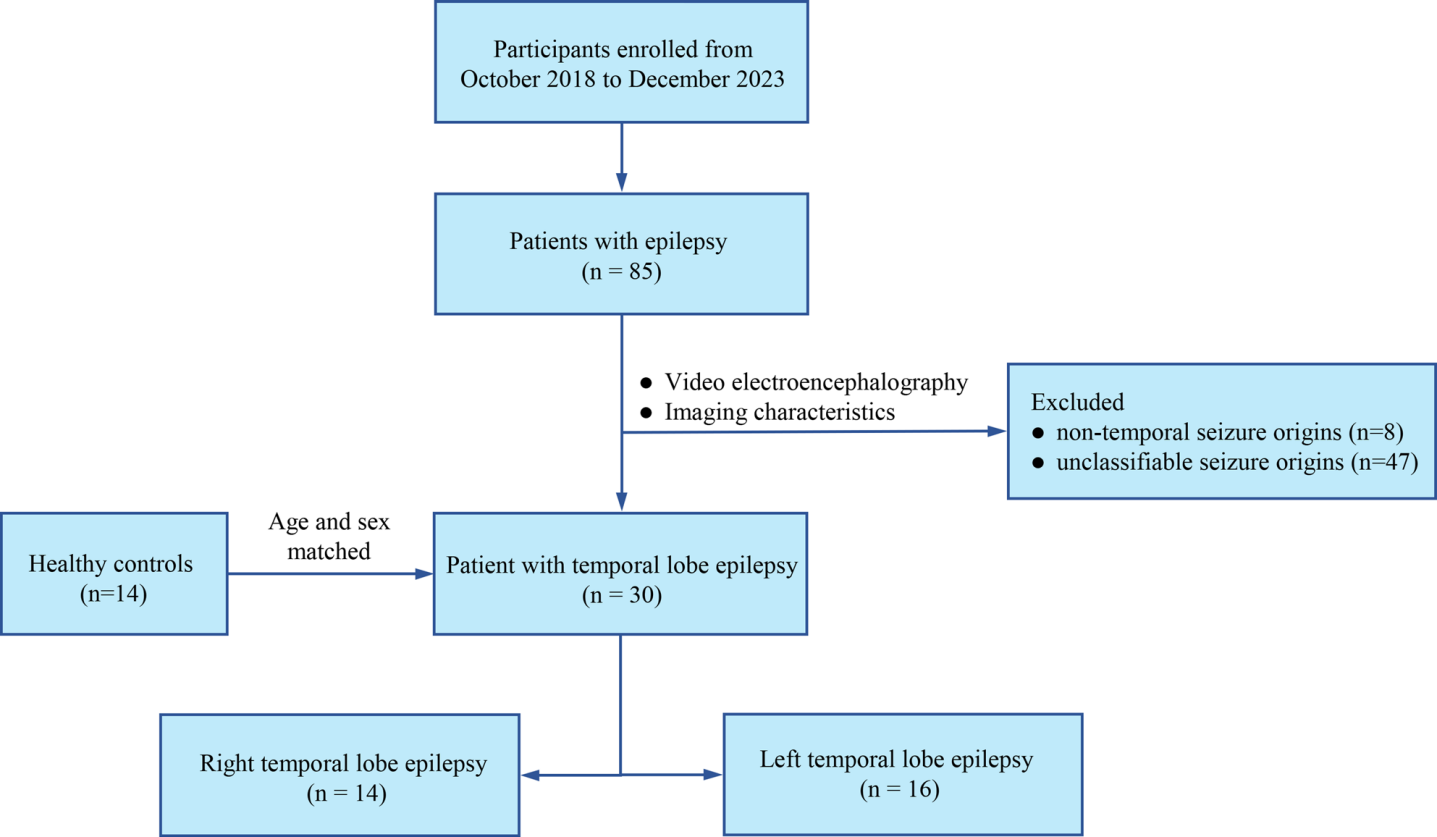
Screening process of participant enrollment

The retrospective cohort study consecutively enrolled 85 adult patients diagnosed with epilepsy at the epilepsy centre of our hospital between October 2018 and December 2023. All participants satisfied the diagnostic criteria established by the Commission on Classification and Terminology of the International League Against Epilepsy. Exclusion criteria comprised: (1) bilateral epileptogenic foci; (2) non-temporal or unclassifiable seizure origins; and (3) poor image quality. Following rigorous screening, 8 patients were excluded due to non-temporal lobe origin, and 47 were excluded because the seizure onset zone could not be determined. Ultimately, 30 patients with unilateral TLE were selected. Demographic and clinical characteristics, including age, sex, epilepsy onset age, disease duration, and seizure frequency were recorded. Patients were categorised into LTLE (*n* = 16) and RTLE (*n* = 14) groups based on their video electroencephalography, and imaging characteristics. Two RTLE patients lacked complete records regarding onset age, disease duration, and seizure frequency. Fourteen age- and sex-matched healthy controls underwent identical MRI protocols, with exclusion of individuals exhibiting structural brain abnormalities, neurological or psychiatric disorders. The study protocol adhered to the Declaration of Helsinki (as revised in 2013) and was approved by the Ethics Committee of the Fujian Medical University Union Hospital (No. 2021KJCX055). Informed consent was obtained from all participants. A visual depiction of the screening process is shown in Fig. 1.

### Data acquisition

Imaging acquisition was conducted on a 3.0-Tesla MR scanner (MAGNETOM Prisma, Siemens Healthineers, Erlangen, Germany) utilizing a 64-channel receive head coil. The standardised imaging protocols comprised sagittal T1-weighted magnetization prepared rapid gradient-echo (T1-MPRAGE), axial T2-weighted fluid-attenuated inversion recovery (T2-FLAIR), and diffusion MRI sequences. (1) The sagittal T1-MPRAGE sequence parameters were as follows: repetition time (TR) = 2300 ms; echo time (TE) = 2.32 ms; inversion time (TI) = 900 ms; data matrix = 256 × 256; field of view (FOV) = 240 × 240 mm^2^; voxel size = 0.9375 × 0.9375 × 0.9 mm³; and number of averages = 1. (2) T2-FLAIR, FOV = 230 × 201 mm^2^, matrix = 256 × 224, echo train length = 28, slice thickness = 3 mm, TR = 10,380 ms, TE = 95 ms, inversion time = 2570 ms, bandwidth = 235 Hz. (3) Diffusion MRI of 128 q-space samples was acquired using a spinning-echo echo-planar imaging (SE-EPI) sequence which consisted of 21 b-values of 200, 350, 400, 550, 750, 950, 1100, 1150, 1500, 1650, 1700, 1850, 1900, 2050, 2250, 2400, 2450, 2600, 2650, 2950 and 3000 s/mm^2^ along 1, 3, 2, 4, 4, 3, 12, 8, 4, 6, 1, 14, 8, 4, 12, 4, 4, 8, 20, 4, 1 and 2 directions, respectively. TR = 3400 ms; TE = 87 ms; FOV = 230 × 230 mm^2^; Generalized Autocalibrating Partial Parallel Acquisition (GRAPPA) = 2; slice acceleration factor = 2; number of averages = 1; voxel size = 2.5 × 2.5 × 2.5 mm^3^, without gap; bandwidth = 2590 Hz; pulse = bipolar.

### Quantification of DTI-ALPS index and DKI-ALPS index

The DTI-ALPS and DKI-ALPS indices were calculated using a method that is consistent with prior reports [19]. Diffusion-weighted images underwent preprocessing via QSIPrep 0.17.0 (https://qsiprep.readthedocs.io/), incorporating denoising and motion correction. We calculated the diffusivities and kurtosis maps along the x-, y-, and z-axes using Diffusion Imaging in Python (DIPY) software package (https://dipy.org/). Concurrently diffusion and kurtosis maps were spatially normalised to MNI space using ANTs (https://github.com/ANTsX/ANTs) via fractional anisotropy (FA) images, followed by manual visual inspection of registration accuracy. Four 3-mm-diameter spherical regions of interest (ROIs) were manually placed in the projection and association fibre regions of bilateral hemispheres using JHU-ICBM-FA template on colour-coded FA images. The principal fibres of projection fibre regions extended along the z-axis, while those in association fibre regions extended along the y-axis. This process was performed in ITK-SNAP (https://www.itksnap.org/) by two radiologists, with seven and two years of experience respectively, who were blinded to clinical data, ensuring ROIs avoided white matter hyperintensity regions. The diffusion diffusivities and kurtosis along three orthogonal axes were extracted from each ROI. The DTI-ALPS and DKI-ALPS indices were separately calculated for each hemisphere using the following formula.

1 $$DTI-ALPS{\text{ }}index{\text{ = }}\frac{{mean(Dxxproj,Dxxassoc)}}{{mean(Dyyproj,Dzzassoc)}}$$

2 $$DKI-ALPS{\text{ }}index{\text{ = }}\frac{{mean(Kxxxxproj,Kxxxxassoc)}}{{mean(Kyyyyproj,Kzzzzassoc)}}$$

**Fig. 2:**
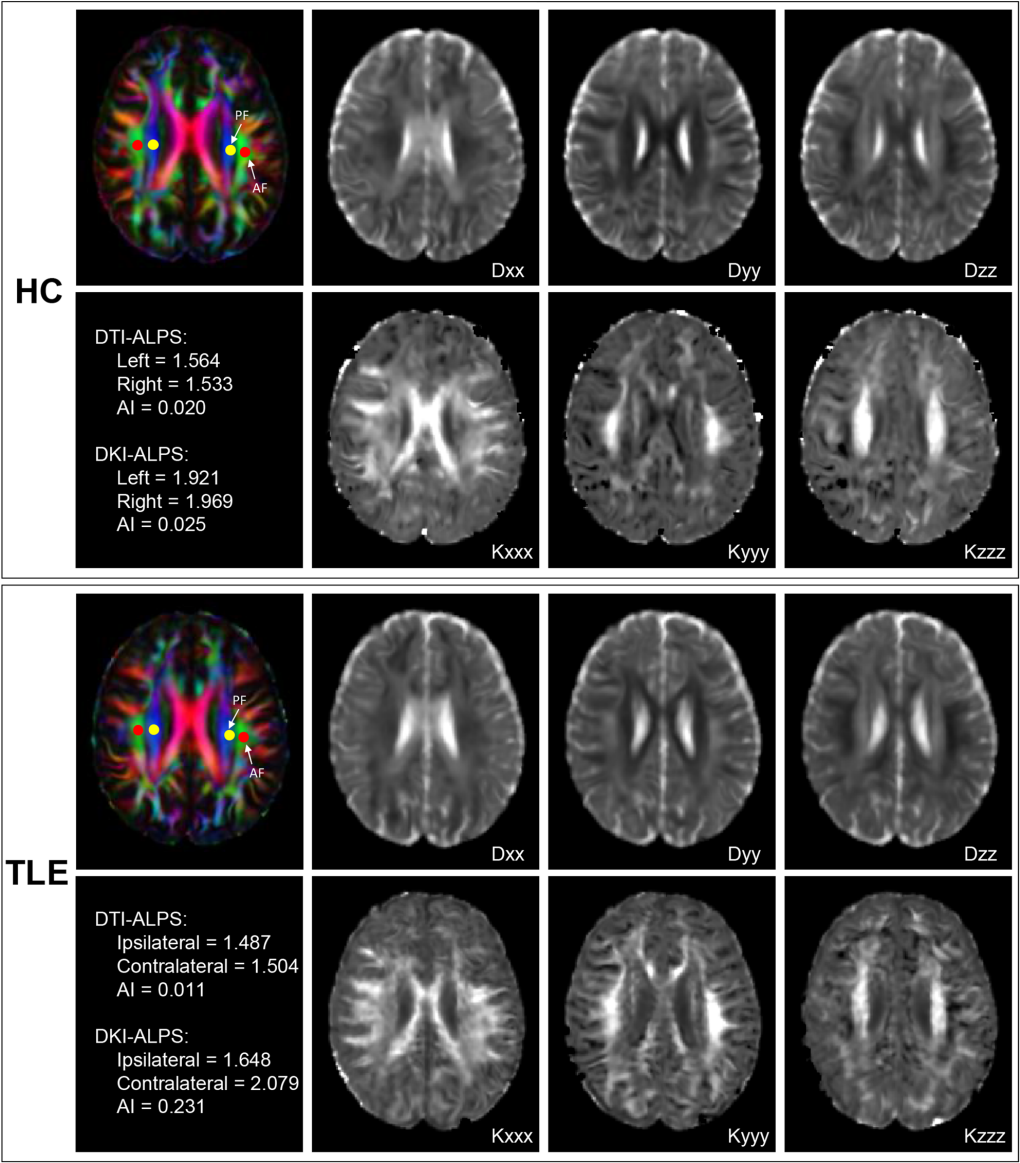
Processed diffusion imaging for the representative HC participant and LTLE patient. We calculated the diffusivities and kurtosis maps along the x-, y-, and z-axes. Concurrently diffusion and kurtosis maps were spatially normalised to MNI space via FA images, followed by manual visual inspection of registration accuracy. Four 3-mm-diameter spherical regions of interest were manually placed in the projection and association fibre regions of bilateral hemispheres using JHU-ICBM-FA template on colour-coded FA images. Compared to the HC participant, the LTLE patient showed reduced ALPS indices on the ipsilateral side, with increased asymmetry on DKI-ALPS. HC, healthy control; LTLE, left temporal lobe epilepsy; FA, fractional anisotropy; TLE, temporal lobe epilepsy; PF, projection fibre; AF, association fibre; DTI-ALPS, diffusion tensor imaging analysis along the perivascular space; DKI-ALPS, diffusion kurtosis imaging analysis along the perivascular space; AI, asymmetry index

Where Dxxproj and Kxxxxproj represented the diffusivities and kurtosis along the x-axis in the projection fibre, Dxxassoc and Kxxxxassoc represented the diffusivities and kurtosis along the x-axis in the association fibre, Dyyproj and Kyyyyproj represented the diffusivities and kurtosis along the y-axis in the projection fibre, and Dzzassoc and Kzzzzassoc represented the diffusivities and kurtosis along the z-axis in the association fibre. Processed diffusion imaging for the representative HC participant and TLE patient are depicted in Fig. 2.

### Morphological analysis

For all participants, automated segmentation of the choroid plexuses was performed on T1-weighted images using a deep learning method with 3D U-NET architecture, which had demonstrated improved segmentation accuracy compared to commercially available algorithms [22]. Brain structures were automatically segmented through the “recon all” pipeline of FreeSurfer 7.2.0 (https://surfer.nmr.mgh.harvard.edu/) to obtain total intracranial volume (TIV), hippocampal volume (HV) and LVV. Absolute volumes of target structures were standardised against TIV.

3 $$Standardised{\text{ }}volume{\text{ = }}\frac{{Absolute{\text{ }}volume}}{{TIV}} \times 100\% $$

**Fig. 3:**
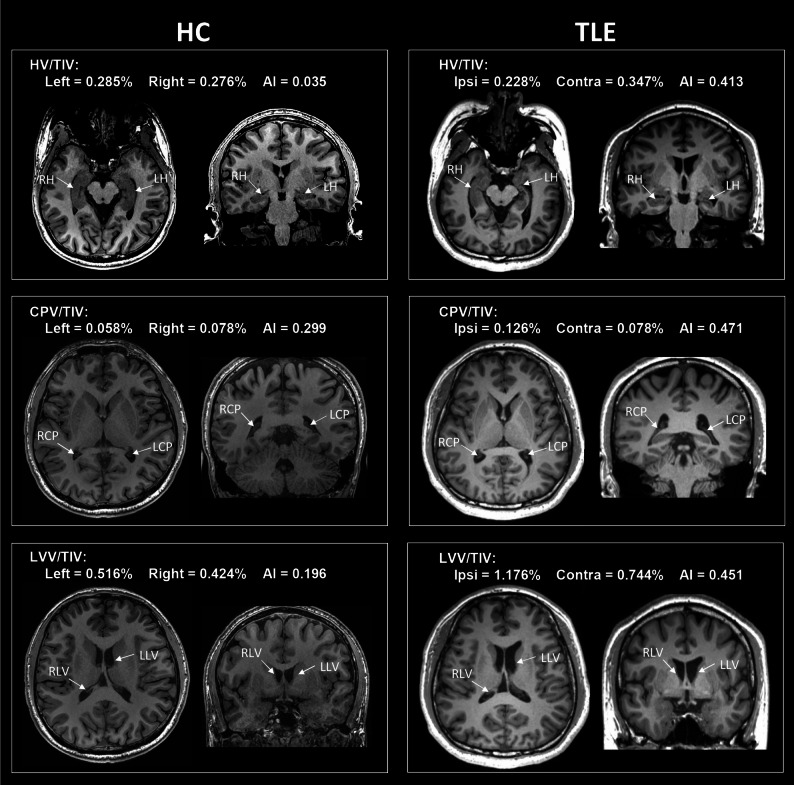
Processed structural imaging for the representative HC participant and LTLE patient. Compared to the HC participant, the LTLE patient showed reduced HV/TIV on the ipsilateral side with increased HV/TIV on the contralateral side, resulting in a higher AI. Additionally, CPV/TIV was increased on the ipsilateral side, contributing to a greater AI. LVV/TIV were increased bilaterally, and this increase was more pronounced on the ipsilateral side, leading to an elevated AI. HC, healthy control; LTLE, left temporal lobe epilepsy; TLE, temporal lobe epilepsy; TIV, total intracranial volume; HV, hippocampal volume; CPV, choroid plexus volume; LVV, lateral ventricular volume; AI, asymmetry index; LCP, left choroid plexus; RCP, right choroid plexus; LH, left hippocampus; RH, right hippocampus; LLV, left lateral ventricle; RLV, right lateral ventricle; Ipsi, ipsilateral; Contra, contralateral

Subsequently, the standardised volumes, including HV/TIV, CPV/TIV, and LVV/TIV, were incorporated into statistical analyses. Processed structural imaging for the representative HC participant and TLE patient are depicted in Fig. 3.

### Quantification of asymmetry index

Interhemispheric asymmetry was quantified using the asymmetry index (AI):

4 $$AI=\frac{{ABS(Right - Left)}}{{mean(Right,Left)}} \times 100\% $$

Where right and left represented the corresponding hemispheric imaging characteristics, with ABS representing the absolute value operator. Subsequently, AIs were derived for five imaging characteristics: AI of HV/TIV, CPV/TIV, LVV/TIV, DTI-ALPS index, and DKI-ALPS index.

### Statistical analyses

Categorical and continuous variables were presented as frequencies (percentages) and mean ± standard deviation (SD), respectively. Given that the ROI placement is operator-dependent, a reproducibility analysis was conducted to assess the robustness of the method. Two raters with differing levels of experience in ROI placement participated in this analysis. Inter-rater reliability was evaluated using the intraclass correlation coefficient (ICC), employing a two-way random model with single measures for absolute agreement.

Given the absence of significant interhemispheric differences in imaging characteristics within the HC group, the average values from bilateral hemispheres were subsequently calculated as baseline measures for between-participant comparisons. The normality of all continuous variables was determined by the Shapiro-Wilk test. For normally distributed data, within-participant comparisons utilised paired t-tests, while between-participant comparisons used independent t-tests or one-way ANOVA. Where the latter revealed significant differences, Bonferroni post-hoc testing was conducted. Accordingly, for data violating the normality assumption, the corresponding analyses were the Wilcoxon signed-rank test for within-participant comparisons and the Mann-Whitney U or Kruskal-Wallis test for between-participant comparisons. Subsequently, significant between-participant results were subjected to pairwise comparisons with Bonferroni correction. The significance of categorical variables was analysed using Pearson’s chi-square tests.

In the analysis of the impairment lateralisation in the brain waste clearance network, a 2 × 2 mixed-model ANOVA was employed, with group (LTLE, RTLE) as the between-subjects factor and lateralisation of the relevant metric (left, right) as the within-subject factor. Subsequently, paired t-tests were applied to examine the effects of LTLE and RTLE on the metric values from the left and right sides.

Spearman’s rank partial correlation analyses were performed, controlling for age, to evaluate associations. The significance threshold was set at *p* < 0.05 (two-tailed). All statistical analyses were conducted using the software SPSS version 27.0, GraphPad Prism version 10.1.2, and R version 4.4.2.

## Results

### Demographic and clinical characteristics

This study recruited 30 adult patients with unilateral TLE (18 males and 12 females; mean age ± SD: 30.533 ± 7.454 years), comprising 16 LTLE cases (11 males and 5 females; mean age ± SD: 30.125 ± 7.032 years) and 14 RTLE cases (7 males and 7 females; mean age ± SD: 31.000 ± 8.152 years). Additionally, 14 HCs (7 males and 7 females; mean age ± SD: 29.857 ± 6.087 years) were enrolled. No significant differences in age or gender distribution were observed among the three groups. Clinical characteristics showed no significant differences between LTLE and RTLE cohorts, including epilepsy onset age (*p* = 0.864), disease duration (*p* = 0.940), and seizure frequency (*p* = 0.873). Table 1 summarised the demographic and clinical characteristics of all participants, with patient data stratified by epileptogenic lateralisation.

**Table 1:** Demographic and clinical characteristics

	TLE			HC (*n* = 14)	*p* value
	Total (*n* = 30)	Left (*n* = 16)	Right (*n* = 14)		
Age (years)	30.533 ± 7.454	30.125 ± 7.032	31.000 ± 8.152	29.857 ± 6.087	0.906^b^
Gender (male/female)	18/12	11/5	7/7	7/7	0.482^c^
Onset age (years)^a^	18.286 ± 11.741	18.625 ± 10.794	17.833 ± 13.381	NA	0.864^d^
Duration (years)^a^	11.693 ± 9.534	11.572 ± 10.237	11.854 ± 8.953	NA	0.940^d^
Seizure frequency (per year)^a^	45.268 ± 77.807	53.219 ± 94.464	34.667 ± 49.767	NA	0.873^e^

Data were presented as mean±standard deviation

^a^Two right temporal lobe epilepsy patients lacked complete records regarding onset age, disease duration, and seizure frequency

^b^One-way ANOVA among the LTLE, RTLE and HC groups

^c^Chi-square test among the LTLE, RTLE and HC groups

^d^Two-sample t-test between the LTLE and RTLE groups

^e^Mann-Whitney U test between the LTLE and RTLE groups

TLE, temporal lobe epilepsy; LTLE, left temporal lobe epilepsy; RTLE, right temporal lobe epilepsy; HC, healthy control; NA, not applicable

### Reproducibility analysis

Two evaluators with differing levels of expertise in ROI placement contributed to this assessment. The overall ICC for ALPS values was 0.939 (95% confidence interval: 0.918–0.954, *p* < 0.001). In detail, the ICC for DTI-ALPS attained 0.973 (95% confidence interval: 0.954–0.984) within the TLE cohort and 0.915 (95% confidence interval: 0.824–0.960) within the HC cohort. Correspondingly, the ICC for DKI-ALPS was 0.899 (95% confidence interval: 0.834–0.939) in the TLE group and 0.871 (95% confidence interval: 0.739–0.938) in the HC group, collectively reflecting near-perfect reproducibility of the ALPS index. As the first rater held more substantial neuroimaging qualifications, the ALPS values derived by this rater were employed as the baseline dataset in subsequent statistical analyses.

### Within-participant analysis

Table 2 and Supplemental Figure S1 present the results of the paired comparisons for the characteristics between the ipsilateral and contralateral sides in the TLE, LTLE and RTLE groups, and between the left and right sides in the HC group. Compared to the contralateral side, the TLE group showed significantly decreased HV/TIV (*p* < 0.001), DTI-ALPS index (*p* = 0.008), and DKI-ALPS index (*p* = 0.024) in the ipsilateral hemisphere; however, CPV/TIV (*p* = 0.678) and LVV/TIV (*p* = 0.289) showed no significant differences between the ipsilateral and contralateral sides. In the LTLE group, the ipsilateral side demonstrated significantly decreased HV/TIV (*p* = 0.003) and increased LVV/TIV (*p* = 0.020) compared to the contralateral side; no significant differences were observed between both sides for CPV/TIV, DTI-ALPS index, or DKI-ALPS index (*p* = 0.411, 0.394, and 0.326, respectively). Consistent with the TLE group, the RTLE group exhibited a significant decrease in HV/TIV (*p* = 0.034), DTI-ALPS index (*p* = 0.006), and DKI-ALPS index (*p* = 0.009) on the ipsilateral side compared to the contralateral side; however, no interhemispheric differences were detected in CPV/TIV and LVV/TIV (*p* = 0.171 and *p* = 0.127 respectively). In addition, no significant difference was observed in any structural or diffusion imaging characteristics within the HC group’s bilateral hemispheres.

**Table 2:** Within-participant comparisons of structural and diffusion imaging characteristics

	Structural imaging characteristics				Diffusion imaging characteristics	
	HV/TIV (%)	CPV/TIV (%)	LVV/TIV (%)		DTI-ALPS index	DKI-ALPS index
**TLE (n = 30)**						
Ipsilateral	0.277 ± 0.062	0.084 ± 0.019	0.979 ± 0.877		1.475 ± 0.187	1.850 ± 0.362
Contralateral	0.334 ± 0.033	0.086 ± 0.023	0.879 ± 0.619		1.566 ± 0.191	2.095 ± 0.446
*p* value	<0.001^a^	0.678^a^	0.289^b^		0.008^b^	0.024^b^
**LTLE (n = 16)**						
Ipsilateral	0.256 ± 0.051	0.091 ± 0.020	1.235 ± 1.152		1.434 ± 0.200	1.830 ± 0.408
Contralateral	0.324 ± 0.031	0.086 ± 0.025	1.014 ± 0.822		1.475 ± 0.097	2.042 ± 0.488
*p* value	0.003^b^	0.411^a^	0.020^b^		0.394^a^	0.326^b^
**RTLE (n = 14)**						
Ipsilateral	0.302 ± 0.067	0.076 ± 0.013	0.686 ± 0.109		1.521 ± 0.167	1.873 ± 0.315
Contralateral	0.346 ± 0.032	0.084 ± 0.021	0.725 ± 0.165		1.671 ± 0.219	2.156 ± 0.402
*p* value	0.034^a^	0.171^a^	0.127^a^		0.006^a^	0.009^b^
**HC (n = 14)**						
Left	0.315 ± 0.015	0.064 ± 0.027	0.626 ± 0.176		1.642 ± 0.128	2.064 ± 0.236
Right	0.318 ± 0.018	0.078 ± 0.017	0.584 ± 0.198		1.618 ± 0.142	2.142 ± 0.253
*p* value	0.250^a^	0.058^a^	0.084^b^		0.470^b^	0.084^b^

Data were presented as mean±standard deviation

^a^Paired t-test

^b^Wilcoxon signed-rank test

TIV, total intracranial volume; HV, hippocampal volume; CPV, choroid plexus volume; LVV, lateral ventricular volume; DTI-ALPS, diffusion tensor imaging analysis along the perivascular space; DKI-ALPS, diffusion kurtosis imaging analysis along the perivascular space; TLE, temporal lobe epilepsy; LTLE, left temporal lobe epilepsy; RTLE, right temporal lobe epilepsy; HC, healthy control

### Between-participant analysis

**Fig. 4:**
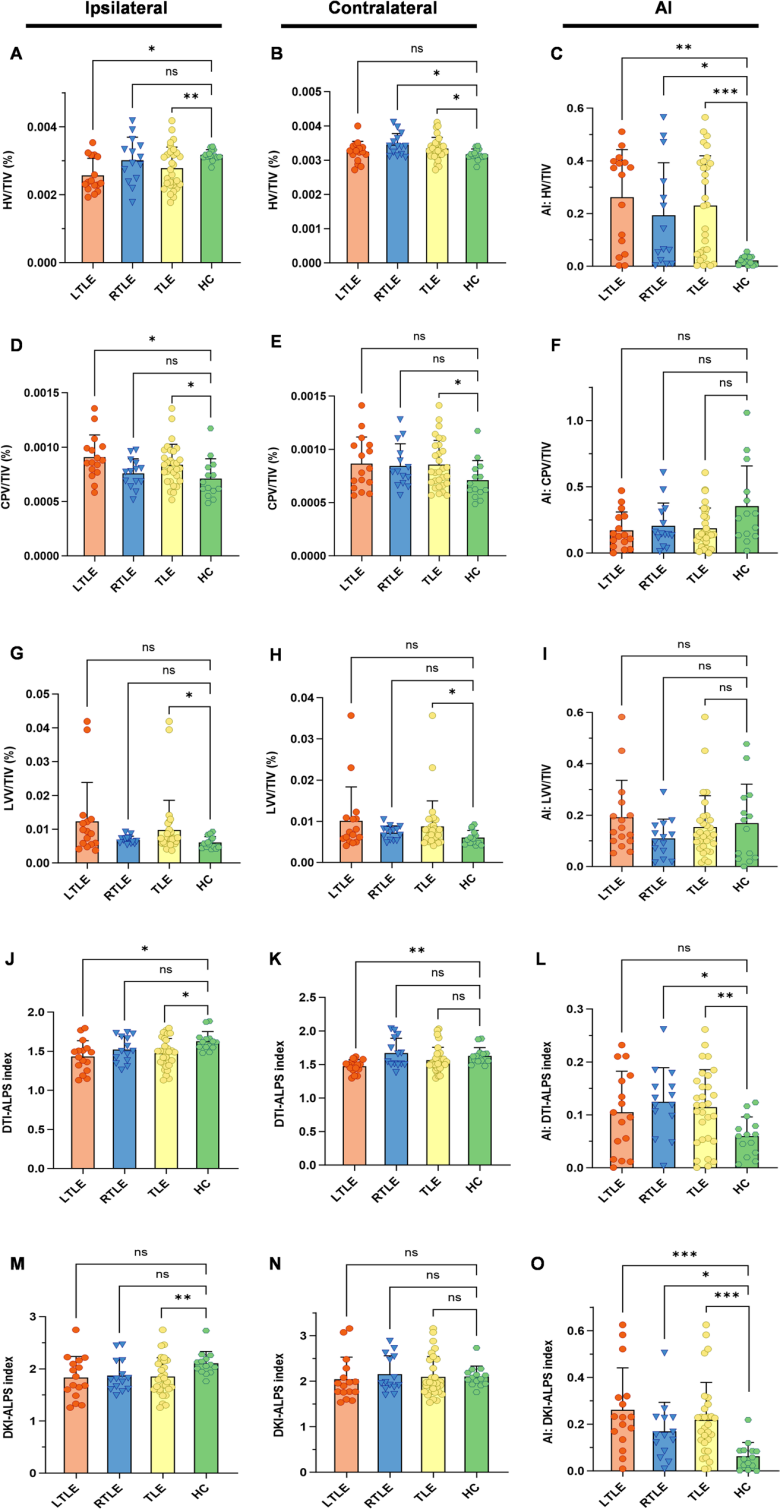
Between-participant comparisons across the TLE, LTLE, RTLE and HC groups. In the TLE group, ipsilateral HV/TIV (**A**) was significantly decreased and contralateral HV/TIV (**B**) was significantly increased compared to the bilateral means of the HC group; AI of HV/TIV (**C**) were significantly higher than that in the HC group; both ipsilateral and contralateral CPV/TIV (**D**, **E**) and LVV/TIV (**G**, **H**) were significantly increased; both ipsilateral DTI-ALPS index (**J**) and DKI-ALPS index (**M**) were significantly decreased; AI of the DTI-ALPS index (**L**) and AI of the DKI-ALPS index (**O**) were significantly higher than those in the HC group. In the LTLE group, ipsilateral HV/TIV was significantly decreased compared to the bilateral means of the HC group (**A**); AI of HV/TIV was significantly higher than that in the HC group (**C**); ipsilateral CPV/TIV (**D**) was significantly increased; both ipsilateral and contralateral DTI-ALPS indices were significantly reduced (**J**, **K**); AI of the DKI-ALPS index in LTLE patients was significantly higher than that in HC participants (**O**). In the RTLE group, contralateral HV/TIV was significantly increased compared to the bilateral means of the HC group (**B**); AI of HV/TIV was significantly higher than that in the HC group (**C**); AI of the DTI-ALPS index and AI of the DKI-ALPS index were both significantly increased compared to the HC group (**L** and **O**). *, *p* < 0.05; **, *p* < 0.01; ***, *p* < 0.001; ns, not significant. TLE, temporal lobe epilepsy; LTLE, left temporal lobe epilepsy; RTLE, right temporal lobe epilepsy; HC, healthy control; TIV, total intracranial volume; HV, hippocampal volume; CPV, choroid plexus volume; LVV, lateral ventricular volume; DTI-ALPS, diffusion tensor imaging analysis along the perivascular space; DKI-ALPS, diffusion kurtosis imaging analysis along the perivascular space; AI, asymmetry index

Table 3 and Fig. 4 present the results of the between-participant comparisons of ipsilateral and contralateral imaging characteristics, and their AIs across the TLE, LTLE, RTLE and HC groups. In the TLE group, for structural imaging characteristics, ipsilateral HV/TIV (*p* = 0.003) was significantly decreased and contralateral HV/TIV (*p* = 0.023) was significantly increased compared to the bilateral means of the HC group; AI of HV/TIV was significantly higher than that in the HC group (*p* < 0.001); both ipsilateral and contralateral CPV/TIV (*p* = 0.039 and *p* = 0.042 respectively) and LVV/TIV (*p* = 0.030 and *p* = 0.032 respectively) were significantly increased. For diffusion imaging characteristics, both the ipsilateral DTI-ALPS index and DKI-ALPS index were significantly decreased (*p* = 0.011 and *p* = 0.008 respectively), while their respective AIs were significantly higher than those in the HC group (*p* = 0.002 and *p* < 0.001 respectively).

**Table 3:** Between-participant comparisons of structural and diffusion imaging characteristics

	TLE			HC (*n* = 14)	*p* value		
	Total (*n* = 30)	Left (*n* = 16)	Right (*n* = 14)		A	B	C/D/E
**Structural imaging characteristics**							
HV/TIV							
Ipsilateral (%)	0.277 ± 0.062	0.256 ± 0.051	0.302 ± 0.067	0.317 ± 0.016	0.003^a^	0.016^b^	0.018/1.000/0.131^c^
Contralateral (%)	0.334 ± 0.033	0.324 ± 0.031	0.346 ± 0.032		0.023^a^	0.022^d^	1.000/0.025/0.117^c^
AI	0.230 ± 0.190	0.262 ± 0.181	0.193 ± 0.200	0.023 ± 0.017	<0.001^e^	0.001^b^	0.001/0.029/1.000^c^
CPV/TIV							
Ipsilateral (%)	0.084 ± 0.019	0.091 ± 0.020	0.076 ± 0.013	0.071 ± 0.018	0.039^a^	0.010^d^	0.012/1.000/0.082^c^
Contralateral (%)	0.086 ± 0.023	0.086 ± 0.025	0.084 ± 0.021		0.042^a^	0.127^d^	NA
AI	0.187 ± 0.153	0.170 ± 0.139	0.206 ± 0.171	0.355 ± 0.302	0.062^e^	0.143^b^	NA
LVV/TIV							
Ipsilateral (%)	0.979 ± 0.877	1.235 ± 1.152	0.686 ± 0.109	0.605 ± 0.175	0.030^e^	0.056^b^	NA
Contralateral (%)	0.879 ± 0.619	1.014 ± 0.822	0.725 ± 0.165		0.032^e^	0.097^b^	NA
AI	0.154 ± 0.123	0.191 ± 0.145	0.110 ± 0.075	0.169 ± 0.152	0.940^e^	0.225^b^	NA
**Diffusion imaging characteristics**							
DTI-ALPS index							
Ipsilateral	1.475 ± 0.187	1.434 ± 0.200	1.521 ± 0.167	1.630 ± 0.122	0.011^e^	0.019^b^	0.015/0.357/0.690^c^
Contralateral	1.566 ± 0.191	1.475 ± 0.097	1.671 ± 0.219		0.059^e^	0.003^b^	0.008/1.000/0.014^c^
AI	0.114 ± 0.071	0.105 ± 0.077	0.125 ± 0.065	0.060 ± 0.037	0.002^a^	0.027^d^	0.165/0.027/1.000^c^
DKI-ALPS index							
Ipsilateral	1.850 ± 0.362	1.830 ± 0.408	1.873 ± 0.315	2.103 ± 0.230	0.008^a^	0.069^d^	NA
Contralateral	2.095 ± 0.446	2.042 ± 0.488	2.156 ± 0.402		0.257^e^	0.338^b^	NA
AI	0.218 ± 0.160	0.261 ± 0.180	0.169 ± 0.124	0.063 ± 0.059	<0.001^e^	<0.001^b^	<0.001/0.044/0.437^c^

Data were presented as mean±standard deviation

^a^Two-sample t-test

^b^Kruskal-Wallis test among the LTLE, RTLE and HC groups

^c^Pairwise comparisons with Bonferroni correction

^d^One-way ANOVA among the LTLE, RTLE and HC groups

^e^Mann-Whitney U test

A, Total vs. HC; B, between-participant comparisons among the Left, Right and HC groups. C, Left vs. HC; D, Right vs. HC; E, Left vs. Right. TLE, temporal lobe epilepsy; HC, healthy control; TIV, total intracranial volume; HV, hippocampal volume; CPV, choroid plexus volume; LVV, lateral ventricular volume; DTI-ALPS, diffusion tensor imaging analysis along the perivascular space; DKI-ALPS, diffusion kurtosis imaging analysis along the perivascular space; AI, asymmetry index; NA, not applicable

In the LTLE group, for structural imaging characteristics, ipsilateral HV/TIV was significantly decreased compared to the bilateral means of the HC group (*p* = 0.018); AI of HV/TIV was significantly higher than that in the HC group (*p* = 0.001); ipsilateral CPV/TIV was significantly increased (*p* = 0.012). For diffusion imaging characteristics, both ipsilateral and contralateral DTI-ALPS indices were significantly reduced (*p* = 0.015 and *p* = 0.008 respectively); AI of the DKI-ALPS index in LTLE patients was significantly higher than that in HC participants (*p* < 0.001).

In the RTLE group, for structural imaging characteristics, contralateral HV/TIV was significantly increased compared to the bilateral means of the HC group (*p* = 0.025), while AI of HV/TIV was significantly higher than that in the HC group (*p* = 0.029). For diffusion imaging characteristics, AI of the DTI-ALPS index and AI of the DKI-ALPS index were both significantly increased compared to the HC group (*p* = 0.027 and *p* = 0.044 respectively).

### Impairment lateralisation in the brain waste clearance network

The DTI‑ALPS index showed a main effect of group (F(1, 56) = 9.105, *p* = 0.004), with RTLE patients exhibiting 0.138 (95% confidence interval: 0.046–0.230) higher values than the LTLE counterparts, but no laterality effect or interaction. For the DKI‑ALPS index, a significant group‑by‑laterality interaction was observed (F(1, 56) = 5.403, *p* = 0.024). Simple‑effects analysis revealed patients with RTLE exhibited 0.325 (95% confidence interval: 0.024–0.627) higher left values than those with LTLE, with no between‑group difference on the right.

Neither CPV/TIV nor LVV/TIV showed a significant interaction or laterality main effect. However, for LVV/TIV, a main effect of group was identified (F(1, 56) = 4.818, *p* = 0.032), with RTLE patients exhibiting 0.004 lower values (95% confidence interval: 0.000–0.008) than LTLE patients.

### Correlation between the structural and diffusion imaging characteristics

Supplemental Figure S2 to S4 detail the partial correlation analyses between the structural and diffusion imaging characteristics, namely, between ipsilateral structural and diffusion imaging metrics, and between the AIs derived from these respective imaging modalities. However, none of these correlations attained statistical significance in our cohort.

### Correlation between the imaging characteristics and the clinical characteristics

**Fig. 5:**
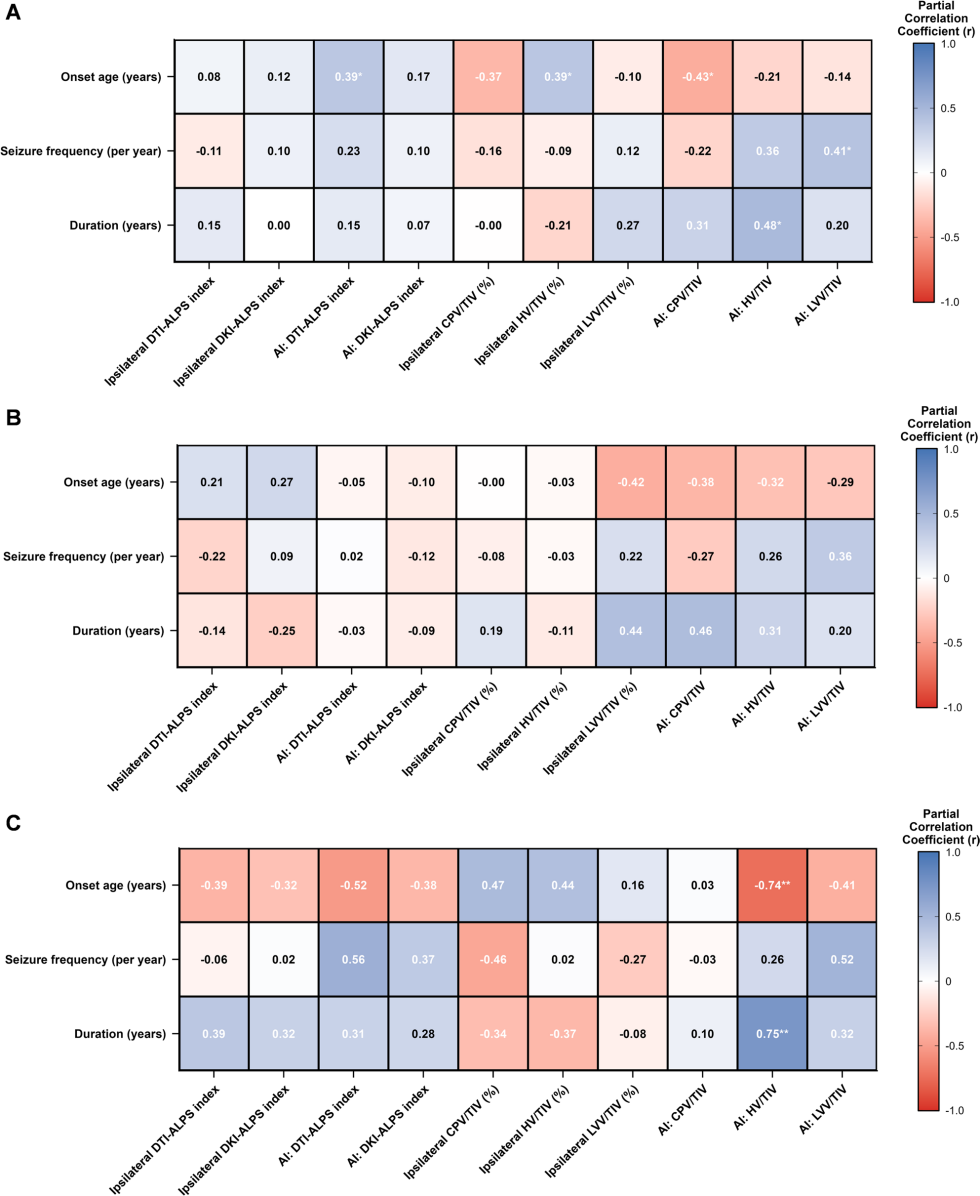
Spearman’s partial correlation heatmap between the imaging characteristics and the clinical characteristics, controlling for age. In the TLE cohort, positive correlations were observed between onset age and both AI of the DTI-ALPS index and ipsilateral HV/TIV; AI of CPV/TIV correlated negatively with onset age; AI of HV/TIV was positively associated with disease duration, and AI of LVV/TIV was positively linked to seizure frequency (**A**). In the LTLE cohort, none of the partial correlations between the imaging characteristics and the clinical characteristics attained statistical significance (**B**). In the RTLE cohort, AI of HV/TIV correlated negatively with onset age and positively with disease duration (**C**). Darker colour intensities correspond to stronger correlations. ALPS, analysis along the perivascular space; TLE, temporal lobe epilepsy; LTLE, left temporal lobe epilepsy; RTLE, right temporal lobe epilepsy; TIV, total intracranial volume; CPV, choroid plexus volume; HV, hippocampal volume; LVV, lateral ventricular volume; DTI-ALPS, diffusion tensor imaging analysis along the perivascular space; DKI-ALPS, diffusion kurtosis imaging analysis along the perivascular space; AI, asymmetry index. *, *p* < 0.05; **, *p* < 0.01; ***, *p* < 0.001

As illustrated in Fig. 5, partial correlation analyses were conducted between the imaging characteristics and the clinical characteristics, controlling for age. Within the RTLE group, AI of HV/TIV correlated negatively with onset age (*r* = -0.740, *p* = 0.009) and positively with disease duration (*r* = 0.745, *p* = 0.008). In the TLE cohort, positive correlations were observed between onset age and both AI of the DTI-ALPS index (*r* = 0.386, *p* = 0.047) and ipsilateral HV/TIV (*r* = 0.392, *p* = 0.043); in contrast, AI of CPV/TIV correlated negatively with onset age (*r* = -0.427, *p* = 0.026). Furthermore, AI of HV/TIV was positively associated with disease duration (*r* = 0.481, *p* = 0.011), and AI of LVV/TIV was positively linked to seizure frequency (*r* = 0.413, *p* = 0.032) in the TLE group. The remaining characteristics did not exhibit any statistically significant correlations.

## Discussion

The principal findings of this study were: (1) TLE patients exhibited a significant reduction in ipsilateral glymphatic function and microstructural complexity, coupled with increased hemispheric asymmetry; (2) beyond ipsilateral hippocampal atrophy and elevated AI of HV/TIV, bilateral enlargement of the choroid plexuses and lateral ventricles was observed; (3) LTLE and RTLE patients might exhibit distinct functional and morphological change patterns of the brain waste clearance network; (4) CPV/TIV showed significant correlations with onset age, with HV/TIV demonstrating correlation with both onset age and disease duration, while LVV/TIV exhibited correlation with seizure frequency.

Consistent with the classical glymphatic MRI with intrathecal gadolinium-based contrast administration, the DTI-ALPS index was also positively correlated with glymphatic clearance function [14, 23]. In our cohort, patients with TLE exhibited decreased bilateral DTI-ALPS indices compared to the bilateral means of the HC group. This reduction was more pronounced ipsilaterally, alongside a significantly increased AI. These findings indicated asymmetrically impaired glymphatic clearance function in TLE patients, aligning with prior studies [15–17]. Clinical data were highly suggestive of loss of selective permeability of blood-brain barrier (BBB) in the focal seizure onset regions [24]. BBB disruption altered water content and movement, impacting ISF dynamics and CSF-ISF exchange. The net results might be failure of interstitial clearance from toxic molecules, such as phosphorylated tau proteins. The recurrent conversion of these toxins into abnormal neuronal activity might further perturb ISF flow, sustaining a pathological vicious cycle [24–26]. Furthermore, Rebecca Emily Feldman et al. demonstrated significantly greater asymmetry in PVS distribution in epilepsy patients versus HC, with maximal asymmetry observed in the suspected seizure onset zone [27]. Thus, we considered our findings robust.

This study found largely concordant reductions in both DTI-ALPS and DKI-ALPS indices in TLE patients, suggesting concurrent alterations in both glymphatic function and its underlying microstructure. However, their biophysical bases differ fundamentally. The DTI-ALPS index serves as an indirect proxy for glymphatic fluid flow by quantifying the rate and directionality of water diffusion. In contrast, the DKI-ALPS index reflects the microstructural complexity and integrity of the perivascular environment by measuring non-Gaussian water diffusion characteristics. Thus, the two indices are complementary: DTI-ALPS informs on the system’s functional efficiency (the “flow”), while DKI-ALPS probes its structural substrate (the “hardware”), such as alterations in the geometry of perivascular spaces, the status of astrocytic endfeet, or extracellular matrix composition. Previous studies have applied the DKI-ALPS index to detect disease-related alterations, which may be attributed to DKI’s capacity to capture microstructural complexities, including fibre crossing present in up to 90% of white matter voxels, scenarios where the DTI model may be limited [19, 28–31]. The observed discrepancy between our findings and the hypothesis that DKI-ALPS is a more sensitive marker may be explained by specific attributes of our clinical cohort. The relatively small sample size compromised statistical power, possibly masking subtle distinctions. Moreover, the enrolled TLE patients had a long average disease duration. Persistent epileptic activity can induce substantial tissue remodelling and glymphatic damage; during chronic stages, both DTI and DKI models can detect significant abnormalities, thereby attenuating any potential difference in sensitivity for detecting gross impairment between the two approaches.

Furthermore, our study found increased CPV/TIV and LVV/TIV in both the ipsilateral and contralateral sides of TLE patients. Considering CSF circulation, which is principally generated by the choroid plexuses, flows through the ventricular system, and drains via the foramina of Magendie and Luschka, together with observations that CP enlargement was associated with glymphatic dysfunction and that CPV positively corresponds to lateral ventricular signal alteration on glymphatic MRI [32], we hypothesised that elevated CPV/TIV and LVV/TIV in TLE patients may indicate impaired cerebral waste clearance, similar to the reduction in ALPS indices. However, our study found no significant correlation between these measures and the ALPS indices. This differed from the findings by JJW et al. [20], who reported a significant negative correlation between CPV/TIV and ipsilateral DTI-ALPS index in their cohort. Notably, the relationship between alterations in the choroid plexuses and those in glymphatic function remained controversial. YFL et al. suggested the possibility of CP abnormality as a positive regulator of glymphatic dysfunction [32], whereas Nicola Marchi et al. found the choroid plexuses are a less relevant place for CSF-ISF exchange [24]. Our limited sample size precluded definitive conclusions on whether choroid plexus or lateral ventricular alterations influenced glymphatic function. Further studies with larger cohorts are needed to validate this relationship.

It is well-established that the hippocampus plays a crucial role in the progression of TLE. Our study observed altered bilateral hippocampal volumes in TLE patients. Specifically, the ipsilateral HV/TIV decreased, while the contralateral HV/TIV increased, resulting in a significantly elevated AI. Ipsilateral hippocampal atrophy is an established feature in TLE patients, which has been consistently reported [33–35]. Additionally, Jae-Gon Yoo et al. found a significant difference in the shape of the left hippocampus of RTLE patients compared to the left hippocampus of controls via morphometrics (shape analysis). They postulated this might arise from functional compensation, abnormal tissue, or a combination of both [34], which was consistent with our findings.

Our study also indicated that functional and structural change patterns within the brain waste clearance network might differ between LTLE and RTLE patients. The 2 × 2 mixed-model ANOVA revealed a significant effect of epileptogenic side on both the ALPS indices and the LVV/TIV. Specifically, the RTLE cohort exhibited comparatively elevated ALPS values, whereas the LTLE cohort demonstrated a relative enlargement of lateral ventricular volume. Furthermore, both within-participant and between-participant comparisons consistently indicated a more bilateral distribution and greater severity of pathological changes in LTLE relative to RTLE, a pattern evident not only in the ALPS indices but also in the CPV/TIV and LVV/TIV. Given that the choroid plexuses, lateral ventricles, and glymphatic system were critical components of the brain waste clearance network, we proposed that LTLE and RTLE patients might exhibit different functional and structural change patterns of the brain waste clearance network. A growing corpus of evidence suggests the network patterns in LTLE and RTLE are not symmetrical entities [36]. The vulnerability to seizure activity in any one part of the network is influenced by activity everywhere else in the network, with hemispheric connectivity differing according to the lateralisation of the temporal lobe of onset [37, 38]. While widespread abnormal interactions of large-scale networks were common feature of both left and right mesial temporal lobe epilepsy (MTLE), patients with left MTLE exhibited weaker interactions among aberrant connections, potentially indicative of an ineffective attempt to restore the functionalities. Conversely, the right MTLE cohort, despite demonstrating more restricted pattern of alterations and a lower average degree of altered connectivity, displayed a higher degree of integration among these changes [38]. This mechanistic distinction might underlie the more profound bihemispheric dysfunction observed in LTLE, in contrast to the predominantly ipsilateral, focal disruptions characteristic of RTLE.

In the analysis of partial correlations between the ipsilateral imaging metrics and clinical characteristics, we observed a significant positive correlation between the ipsilateral HV/TIV and onset age in TLE patients, consistent with the prior research [39]. However, our study did not detect a significant correlation between hippocampal volume and either disease duration or seizure frequency in the TLE cohort. This result contrasted with some previous reports [40, 41]. Asla Pitkänen et al. demonstrated a significant correlation between hippocampal volume and disease duration only in the TLE subgroup experiencing frequent seizures (> 2 seizures per year); this correlation was absent in the rare-seizure subgroup (≤ 2 seizures per year) [40]. Our cohort included 4 patients with seizure frequency below 2 per year (2 LTLE, 2 RTLE). This low-seizure-frequency, combined with the limited sample, might have diminished the statistical power to detect such correlations.

Following age‑adjustment, the analysis of partial correlations between AIs of imaging metrics and clinical characteristics revealed significant correlations in TLE patients. Specifically, a significant correlation was observed between AI of HV/TIV and both onset age and disease duration in RTLE patients. In TLE patients, significant positive correlations were observed between the ipsilateral HV/TIV and onset age, as well as between the AI of HV/TIV and disease duration. These findings align with prior evidence linking younger onset age [39] and longer duration [41] to more pronounced ipsilateral hippocampal atrophy. Notably, a novel negative correlation emerged between AI of CPV/TIV and onset age, supporting our hypothesis and extending prior evidence that CPV increase predicts semantic fluency impairment in TLE [20]. This finding underscores a potential role for the brain waste clearance network in TLE, though large‑scale studies are needed in the future. We also identified a significant positive correlation between AI of LVV/TIV and seizure frequency in TLE patients. Given that the ipsilateral temporal horn of the lateral ventricle expanded progressively with increasing duration in TLE-HS patients, likely due to ongoing medial temporal lobe atrophy [42], the link between greater LVV/TIV asymmetry and higher seizure frequency appears mechanistically justified.

This study has several limitations. Firstly, it is a single-centre retrospective study with a relatively small sample size. Large-scale multicentre studies are needed to further validate our findings. Secondly, participant enrollment involved TLE patients both with and without HS, introducing sample heterogeneity. Thirdly, the ALPS index predominantly reflects the ISF efflux function along the perivenous spaces. It cannot fully characterise the function of CSF influx and CSF-ISF exchange [14]. Moreover, in this study, the DKI-ALPS index was employed as an exploratory biomarker of perivascular microstructural complexity, rather than a direct measure of flow. While this application is based on its sensitivity to non-Gaussian diffusion in complex tissue environments, its specific biophysical correspondence to glymphatic anatomy requires further elucidation. Fourthly, as pharmacotherapy is individually tailored, there is heterogeneity in both the formulations and dosages of anti-seizure medications among the recruited patients with TLE. Consequently, the potential influence of these medications on the brain waste clearance network cannot be ruled out. To mitigate any bias arising from anti-seizure medications, future investigations ought to collect comprehensive data on anti-seizure medication load and incorporate regression analysis.

## Conclusions

Our study demonstrated that beyond hippocampal atrophy, TLE patients exhibited alterations in the brain waste clearance network. Specifically, these included impaired glymphatic function, altered glymphatic microstructure and morphological changes such as enlarged CPV and LVV. LTLE and RTLE might exhibit distinct alteration patterns. Additionally, CPV/TIV showed significant correlations with onset age, with HV/TIV demonstrating correlation with both onset age and disease duration, while LVV/TIV exhibited correlation with seizure frequency. These findings could provide new insights into TLE pathogenesis.

## Supplementary Information

Below is the link to the electronic supplementary material.

Supplementary Material 1

## Data Availability

The datasets used and analysed during the current study are available from the corresponding author upon reasonable request.

## Abbreviations

TLE: Temporal lobe epilepsy
HS: Hippocampal sclerosis
CSF: Cerebrospinal fluid
ISF: Interstitial fluid
PVS: Perivascular spaces
DTI-ALPS: Diffusion tensor imaging analysis along the perivascular space
MRI: Magnetic resonance imaging
HC: Healthy control
LTLE: Left temporal lobe epilepsy
RTLE: Right temporal lobe epilepsy
DKI-ALPS: Diffusion kurtosis imaging analysis along the perivascular space
CP: Choroid plexus
CPV: Choroid plexus volume
LVV: Lateral ventricular volume
FA: Fractional anisotropy
ROI: Region of interest
TIV: Total intracranial volume
HV: Hippocampal volume
AI: Asymmetry index
BBB: Blood-brain barrier
MTLE: Mesial temporal lobe epilepsy
