# Supplementary material for: Alterations in glymphatic system and brain morphology in patients with temporal lobe epilepsy
Source: BMC Med Imaging. 2026 Mar 13;26:205. doi: 10.1186/s12880-026-02279-2 (PMC13101242; doi:10.1186/s12880-026-02279-2)
Supplement: Supplementary file 1 — Supplementary Material 1 [file 12880_2026_2279_MOESM1_ESM.docx]

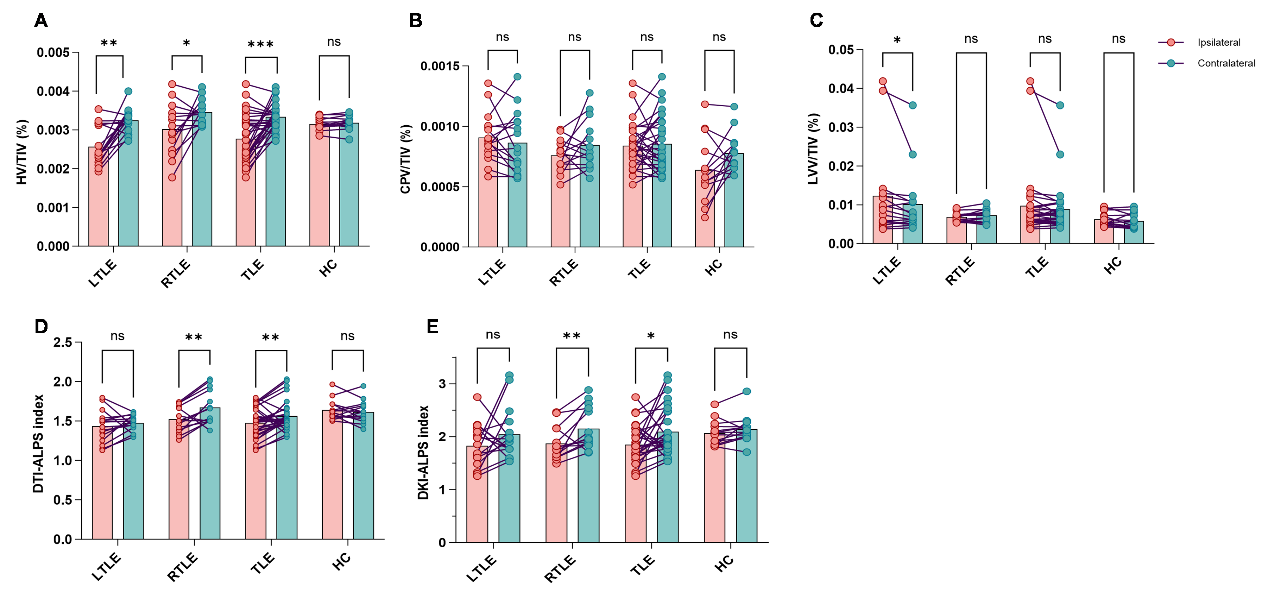


**Supplemental** **Figure S1** Within-participant comparisons for the characteristics between the ipsilateral and contralateral sides in the TLE, LTLE and RTLE groups, and between the left and right sides in the HC group. Compared to the contralateral side, the TLE and RTLE groups showed significantly decreased HV/TIV (A), DTI-ALPS index (D), and DKI-ALPS index (E) in the ipsilateral hemisphere; however, CPV/TIV (B) and LVV/TIV (C) showed no significant differences between the ipsilateral and contralateral sides. In the LTLE group, the ipsilateral side demonstrated significantly decreased HV/TIV (A) and increased LVV/TIV (C) compared to the contralateral side; no significant differences were observed between both sides for CPV/TIV (B), DTI-ALPS index (D), or DKI-ALPS index (E). No significant difference was observed in any structural or diffusion imaging characteristics within the HC group’s bilateral hemispheres (A - E). *, *p* < 0.05; **, *p* < 0.01; ***, *p* < 0.001; ns, not significant. TLE, temporal lobe epilepsy; LTLE, left temporal lobe epilepsy; RTLE, right temporal lobe epilepsy; HC, healthy control; TIV, total intracranial volume; HV, hippocampal volume; CPV, choroid plexus volume; LVV, lateral ventricular volume; DTI-ALPS, diffusion tensor imaging analysis along the perivascular space; DKI-ALPS, diffusion kurtosis imaging analysis along the perivascular space.


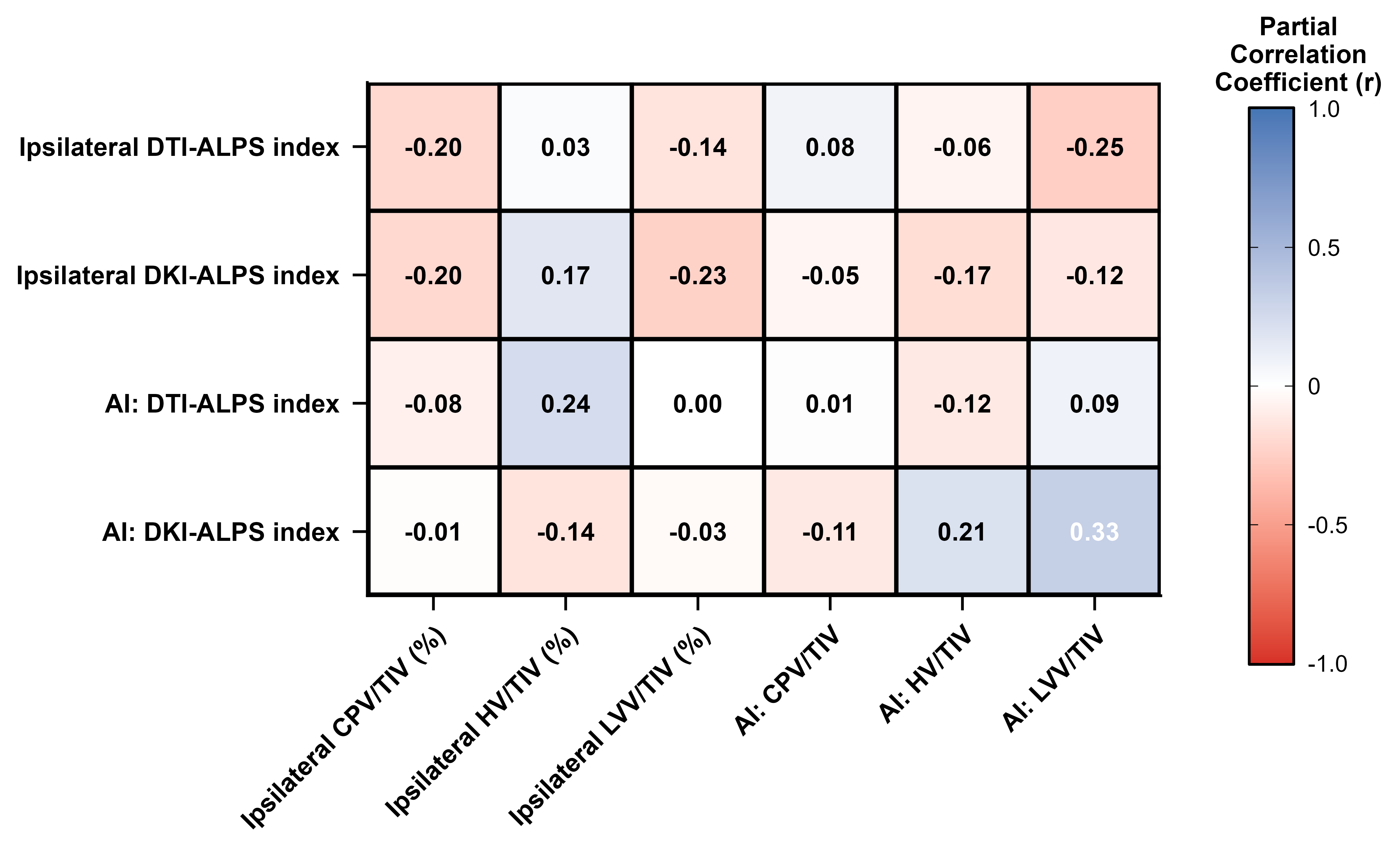


**Supplemental Figure S2** Spearman’s partial correlation heatmap between the ALPS indices and morphological characteristics in patients with TLE, controlling for age. Darker colour intensities correspond to stronger correlations. ALPS, analysis along the perivascular space; TLE, temporal lobe epilepsy; TIV, total intracranial volume; CPV, choroid plexus volume; HV, hippocampal volume; LVV, lateral ventricular volume; DTI-ALPS, diffusion tensor imaging analysis along the perivascular space; DKI-ALPS, diffusion kurtosis imaging analysis along the perivascular space; AI, asymmetry index.


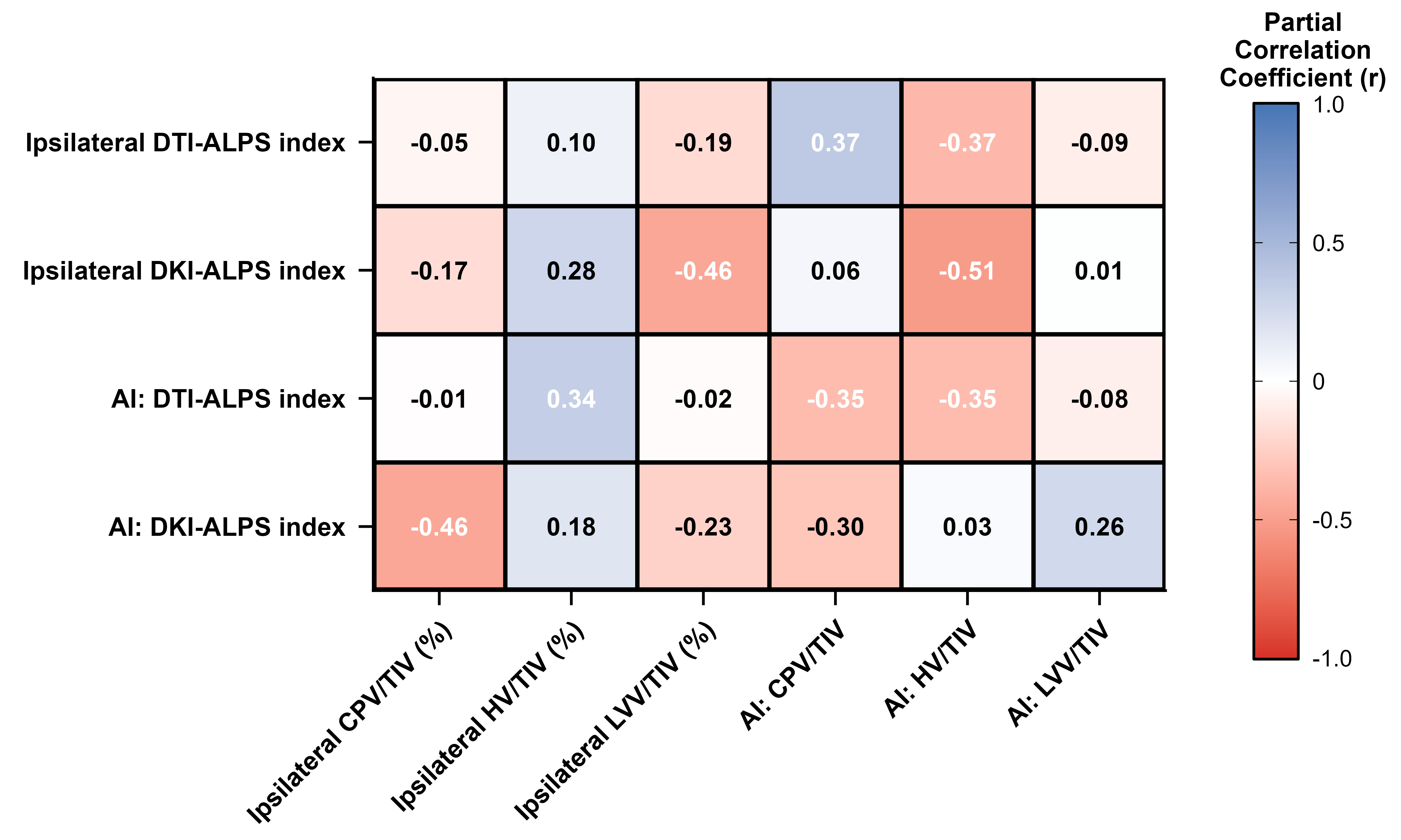


**Supplemental Figure S3** Spearman’s partial correlation heatmap between the ALPS indices and morphological characteristics in patients with LTLE, controlling for age. Darker colour intensities correspond to stronger correlations. ALPS, analysis along the perivascular space; LTLE, left temporal lobe epilepsy; TIV, total intracranial volume; CPV, choroid plexus volume; HV, hippocampal volume; LVV, lateral ventricular volume; DTI-ALPS, diffusion tensor imaging analysis along the perivascular space; DKI-ALPS, diffusion kurtosis imaging analysis along the perivascular space; AI, asymmetry index.


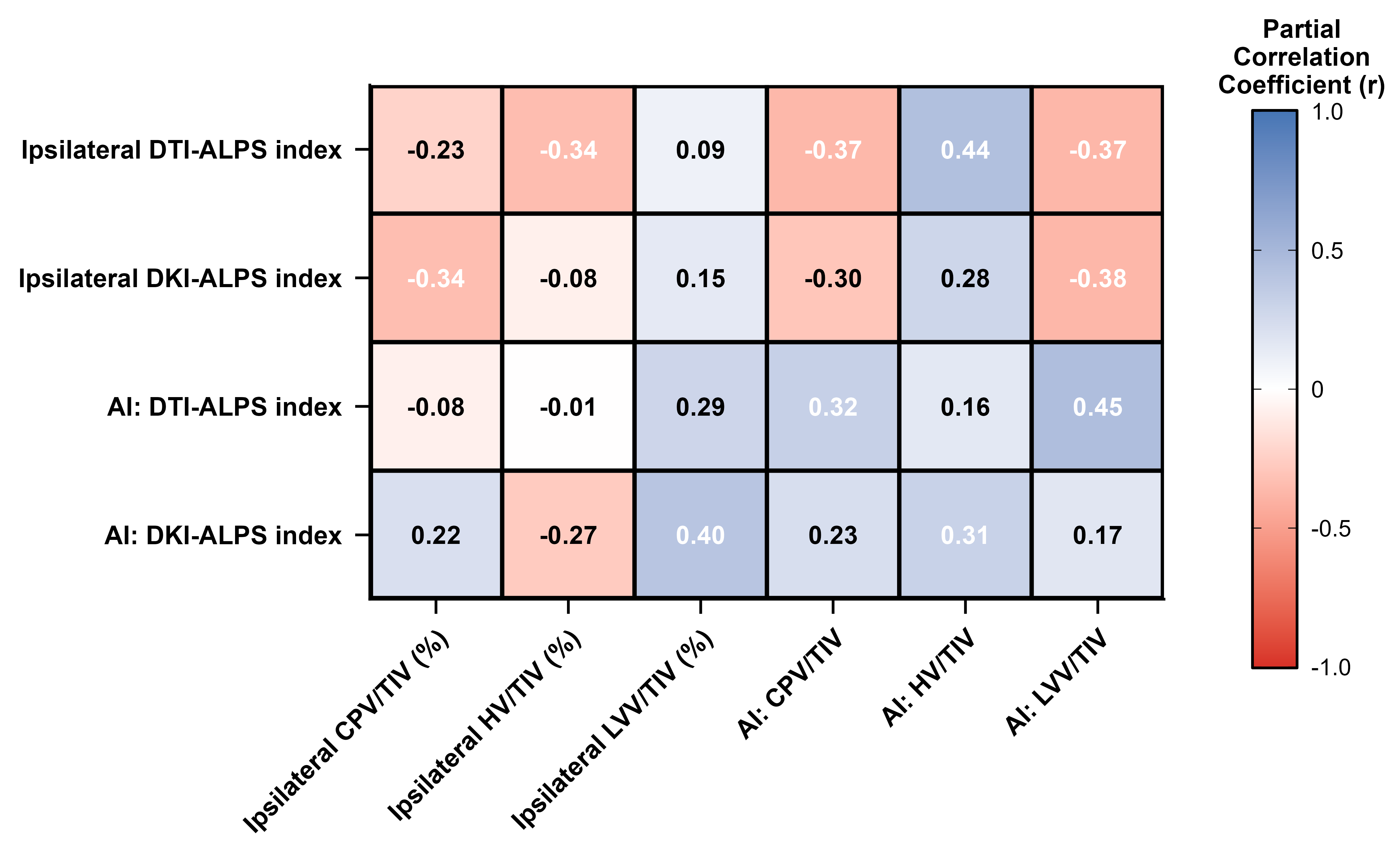


**Supplemental Figure S4** Spearman’s partial correlation heatmap between the ALPS indices and morphological characteristics in patients with RTLE, controlling for age. Darker colour intensities correspond to stronger correlations. ALPS, analysis along the perivascular space; RTLE, right temporal lobe epilepsy; TIV, total intracranial volume; CPV, choroid plexus volume; HV, hippocampal volume; LVV, lateral ventricular volume; DTI-ALPS, diffusion tensor imaging analysis along the perivascular space; DKI-ALPS, diffusion kurtosis imaging analysis along the perivascular space; AI, asymmetry index.
